# Molecular imaging for stem cell therapy in the brain

**DOI:** 10.1186/s13287-015-0242-7

**Published:** 2015-12-18

**Authors:** Nora Sandu, Tumul Chowdhury, Bernhard Schaller

**Affiliations:** University of Oxford, Wellington Square, Oxford, OX1 2JD UK; University of Southampton, University Road, Southampton, SO17 1BJ UK; Department of Anesthesiology and Peri-operative Medicine, University of Manitoba, Winnipeg, MB R3T 2N2 Canada

## Abstract

Molecular imaging is one of the methods to follow-up stem cell therapy by visualization in the brain. In a recent article in *Stem Cell Research & Therapy*, Micci et al. offer a thorough discussion of the advantages and disadvantages of this method and their roles in the future. The authors are among the very first who have implemented recently introduced molecular imaging techniques in experimental research and clinical practice.

## Background

Current neuroimaging techniques give very limited insight into molecular and cellular sequences of events. Therefore, the exact underlying mechanism by which neural stem cells target physiological or pathological brain areas remains elusive. Monitoring of these cells is currently also carried out by the use of various modes of molecular imaging, which is a (novel) technology for visualizing metabolism and signal transduction to gene expression. Most importantly, molecular imaging will render possible the identification of potential therapeutic targets in the development of new treatment strategies and in their successful implementation in clinical application.

## Discussion

In such a context, Micci et al. [1] have demonstrated the human sodium iodide symporter (hNIS), a transmembrane glycoprotein and widely used probe, as a new transporter-based reporter gene for non-invasive molecular imaging and point out one of the principal challenges of molecular imaging of the brain [2–6]. Such new reporter genes will help find the best target on a neurochemical as well as on a commercial level as applying stem cell transplantation in a clinical medium, which is necessity for analysis of the benefit to detect, localize, and examine the stem cells in vivo at both cellular and molecular levels.

Molecular imaging of stem cells has two principal advantages over other methods [6–11]: it allows visual representation, characterization, and quantification of biological processes in the same live recipient over time and it is non-invasive. In this context, hNIS may be an alternative to already-existing vectors like HSV-1 TK or D2R. As hNIS neither is physiologically expressed in the brain nor crosses the intact blood–brain barrier, we can directly examine how it relates to enzymatic activity or gene expression, being perhaps essential to obtain a more detailed description of the failure or success of the cell therapy. Therefore, hNIS incorporated into vectors will make it possible to more fully understand with a relatively high sensitivity the function of protein networks and their role in the spread of stem cells. Such knowledge will facilitate the discovery of still more informative biomarkers.

This restriction leads us to the question of which vector probes should be ideal for molecular imaging of cell therapy. Unfortunately, we think that it is easier to answer this question in different ways. A new vector probe should be able to help to answer some of the ongoing questions in stem cell therapy: the most efficacious route of delivery, the appropriate choice of stem cell type(s), the optimal cell population for treatment in a chronic setting, and the favorable time-point of cell delivery. A safe, non-invasive, and repeatable imaging modality that could identify injected stem cells would be able to answer questions about cell viability and retention as well as provide the ability to adjust the assessment of bioactivity on the basis of actual delivered doses of cells [7, 8]. In the long term, stem cell-derived regeneration still faces difficulties in its efforts to improve because of the need to monitor stem cells continuously with high temporal resolution and good biocompatibility. Under such circumstances, the properties of differentiation and self-renewal of stem cells over long periods of time might be of importance. Moreover, multimodality imaging reporter genes will allow us to choose the imaging technologies that are most appropriate for the biologic problem at hand and facilitate the clinical application of reporter gene technologies.

The investigation by Micci et al. [1] is in line with these current questions of stem cell research, but we have to be aware that stem cell therapy can be used for the clinical daily practice only if safety and efficacy of the transplanted cells can be guaranteed. However, inefficient stem cell differentiation, difficulty in verifying successful delivery to the target organ, and problems with engraftment all hamper the transition from laboratory animal studies to human clinical trials [7–9]. Therefore, there is a need to refine and optimize tracking techniques, as is done by Micci et al. [1]. Moreover, instrumentational improvements, the identification of novel targets and genes, and imaging probe developments suggest that molecular-genetic imaging is likely to play an increasingly important role in the diagnosis and therapy of some brain diseases [11–14]. Fortunately, the advent of molecular imaging will continue to lead unprecedented progress in understanding the fundamental behavior of stem cells, including their survival, bio-distribution, immunogenicity, and tumorogenicity, in the targeted tissues of interest.

## Conclusions

Molecular imaging opens a new door to stem cell therapy, not only in treatment monitoring but also in better understanding the underlying (molecular) processes. Tracking techniques are an important part of this imaging progress and underline the important impact of molecular imaging on patient management with stem cell therapy of the brain.

## Abbreviation

hNIS: human sodium iodide symporter
